# Pedicled Supraclavicular Flap Reconstruction Following Cervicofacial Necrotizing Fasciitis: A Case Report

**DOI:** 10.7759/cureus.109703

**Published:** 2026-05-26

**Authors:** Jorge Flores Orduña, José de Jesús Zapata, Lino Ramirez

**Affiliations:** 1 Surgery, Hospital Regional de Alta Especialidad Centenario de la Revolución Mexicana, Instituto de Seguridad y Servicios Sociales de los Trabajadores del Estado (ISSSTE), Cuernavaca, MEX; 2 General Surgery, Instituto Mexicano del Seguro Social (IMSS) Unidad Médica de Alta Especialidad No. 71, Torreón, MEX; 3 Plastic Surgery, Medical Center Durango Torre V1, Durango, MEX

**Keywords:** cervicofacial infection, deep neck infection, neck reconstruction, necrotizing fasciitis, negative pressure wound therapy, odontogenic infection, pedicled flap, soft tissue infection, supraclavicular flap, vacuum-assisted closure

## Abstract

Cervicofacial necrotizing fasciitis is a life-threatening surgical emergency associated with significant morbidity and mortality, requiring early diagnosis and aggressive multimodal management. Reconstruction of the resulting soft tissue defects represents an additional challenge, particularly in resource-limited settings where microsurgical infrastructure may not be available.

We report the case of a 42-year-old man who developed cervicofacial necrotizing fasciitis secondary to an odontogenic infection. Management consisted of broad-spectrum antibiotic therapy, early surgical debridement, and negative pressure wound therapy as a bridge to reconstruction. Once infection was controlled and a viable granulation tissue bed was established, definitive reconstruction was performed using a pedicled supraclavicular fasciocutaneous flap. The procedure was completed without complications, with satisfactory flap integration, cervicofacial contour restoration, and minimal donor site morbidity documented at outpatient follow-up.

The pedicled supraclavicular flap proved to be a safe, reliable, and technically reproducible reconstructive option in this postinfectious context, offering an intrinsic vascular supply independent of the compromised recipient bed, a wide arc of rotation, and excellent color and texture match to cervicofacial skin. Its applicability in settings without microsurgical resources further supports its role as a first-line reconstructive strategy. This case contributes to the limited evidence on supraclavicular flap reconstruction following cervicofacial necrotizing fasciitis and underscores the value of a sequential, protocol-driven approach in achieving favorable outcomes.

## Introduction

Cervicofacial necrotizing fasciitis is a fulminant soft tissue infection characterized by progressive necrosis of the fascia and subcutaneous tissue, with the potential for spread to the deep cervical spaces and mediastinum. The overall incidence of necrotizing fasciitis ranges from 0.2 to 6.9 cases per 100,000 person-years worldwide, with the cervicofacial region representing a less common site compared to the extremities and perineum [1]. A systematic review of 1,235 cases reported an overall mortality rate of 13.36%, with a mean patient age of 49.1 years and a male predominance of 64.23% [2]. Mortality rises substantially to 41% when descending necrotizing mediastinitis develops, and may reach 64% in the setting of concurrent sepsis; delayed presentation has been identified as an independent adverse prognostic factor [3].

Odontogenic infections constitute the most frequent etiology of cervicofacial necrotizing fasciitis, accounting for 47.04% of reported cases, followed by pharyngolaryngeal (28.34%) and tonsillar or peritonsillar sources (6.07%) [2]. The infectious process spreads through predictable fascial planes of the neck, progressing from the masticatory and submandibular spaces to the parapharyngeal and retropharyngeal spaces, and in its most severe form, to the mediastinum. The infection is predominantly polymicrobial (type I), involving synergistic aerobic and anaerobic microorganisms [4]. The Laboratory Risk Indicator for Necrotizing Fasciitis (LRINEC) score, with a cutoff value ≥ 6, has demonstrated utility as an early diagnostic stratification tool, and its integration with clinical parameters guides timely surgical decision-making [5].

Therapeutic management requires a multimodal approach encompassing broad-spectrum antibiotic therapy covering gram-negative, gram-positive, and anaerobic organisms; aggressive and early surgical debridement; intensive care unit support; and definitive control of the odontogenic infectious source [4]. Negative pressure wound therapy (NPWT) has demonstrated efficacy as an adjunct in the management of complex cervical defects, promoting tissue granulation and reducing bacterial burden within the surgical bed [6]. Alternative reconstructive options in this setting include split-thickness skin grafting, which carries higher rates of graft failure and contraction over poorly vascularized or contaminated beds, and free tissue transfer, which - despite its versatility - requires microvascular infrastructure and prolonged operative time, and is associated with elevated perioperative risk in systemically compromised patients. Reconstruction following extensive cervicofacial debridement poses unique challenges, as the resulting defects are large, irregular, and situated on a contaminated and poorly vascularized bed that limits the reliability of primary closure and conventional grafting techniques. In cases involving extensive soft tissue loss, the pedicled supraclavicular flap constitutes a first-line reconstructive option, based on the supraclavicular artery as an axial pedicle, with a reported complete flap survival rate of 94.2-98.1% [7]. Donor site morbidity is minimal, primary closure is feasible in most cases, and the technique does not require microvascular anastomosis, making it a viable option even in patients with significant comorbidities [8,9]. In the specific context of cervicofacial necrotizing fasciitis, its successful use has been reported for reconstruction of extensive defects of up to 450 cm², with functional and aesthetic outcomes superior to those of the traditional pectoralis major flap [10].

However, published evidence on the use of the supraclavicular flap in the specific context of necrotizing fasciitis remains limited to isolated case reports, and its behavior in a contaminated surgical field with systemic sepsis has not been systematically characterized. We present a case of extensive cervicofacial necrotizing fasciitis secondary to odontogenic infection managed with sequential surgical debridements, negative pressure wound therapy, and pedicled supraclavicular flap reconstruction, with the aim of contributing to the existing evidence on this reconstructive strategy in an infectious context.

## Case presentation

The patient was a 42-year-old male patient, originally from and residing in Durango, Mexico, employed as a security guard, with a secondary-level education and a common-law partnership. His relevant medical history included systemic arterial hypertension with a 27-year diagnostic evolution, managed with losartan 50 mg every 24 hours. Non-pathological personal history included tobacco use with a smoking index of two pack-years, occasional alcohol consumption, and biomass smoke exposure. He had received one dose of the SARS-CoV-2 vaccine. He denied drug allergies, substance abuse, and other comorbidities.

The condition began with pain in the right mandibular region radiating to the ipsilateral ear. The patient initially sought private dental care, where ciprofloxacin was empirically prescribed with a follow-up appointment; however, no clinical improvement was documented. Two days later, mild swelling appeared in the right mandibular region, which progressed during the same day to increased volume in the right cervical region and subsequently the left.

The patient was evaluated at another hospital center, where an exploratory skin incision was performed without drainage of purulent material. Three days after this intervention, he was discharged without imaging studies or surgical specialist evaluation. He subsequently visited a commercial pharmacy clinic, where dicloxacillin and amikacin were prescribed alongside home wound care, without evidence of improvement.

One day later, he presented to the emergency department of our institution with marked anterior cervical swelling, intense pain, and systemic signs of inflammatory response. On admission, leukocytosis of 26,190 cells/mm³ with neutrophilic predominance (neutrophils 83%) was documented, along with hemoglobin 10.0 g/dL, platelets 301,000/mm³, serum sodium 120 mEq/L, glucose 200 mg/dL, creatinine 1.20 mg/dL, CRP 16.4 mg/dL, prothrombin time (PT) 15.9 sec, activated partial thromboplastin time (aPTT) 36.6 sec, and international normalized ratio (INR) 1.21. The LRINEC score was calculated at admission and yielded a total of 7 out of 13 points, corresponding to the intermediate-risk category. Given the suspicion of deep neck infection, consultation with the Oral and Maxillofacial Surgery service was requested.

Two days after admission, surgical drainage of a cervicofacial abscess was performed with abundant purulent output, extraction of tooth #38 as the probable odontogenic source, and floor-of-mouth repair. A Penrose drain was placed, and the patient was transferred to the ICU for advanced management. The following day, bronchial secretion culture reported growth of *Candida albicans*, prompting adjustment of the antimicrobial regimen.

Due to persistence of the infectious process and drain dysfunction, four days after the initial procedure, surgical reintervention was performed with extensive irrigation, debridement of necrotic tissue, and placement of a negative pressure wound therapy/vacuum-assisted closure (NPWT/VAC) system, establishing the definitive diagnosis of bilateral cervicofacial necrotizing fasciitis. The patient was returned to the ICU with orotracheal intubation.

During his ICU stay, the patient required invasive mechanical ventilation and deep sedoanalgesia. Three days after reintervention, the VAC system was exchanged under sedation. Sedation weaning was initiated two days later, with complete neurological recovery achieved 48 hours thereafter.

The following day, the patient was transferred to the hospital ward with a functional VAC system. Mechanical ventilation was successfully discontinued one day later. Two days after transfer to the ward, the VAC system was removed at the bedside, and local wound care with daily Anasept dressings was initiated; however, the patient subsequently developed recurrent purulent drainage, febrile episodes, and chills.

**Figure 1:**
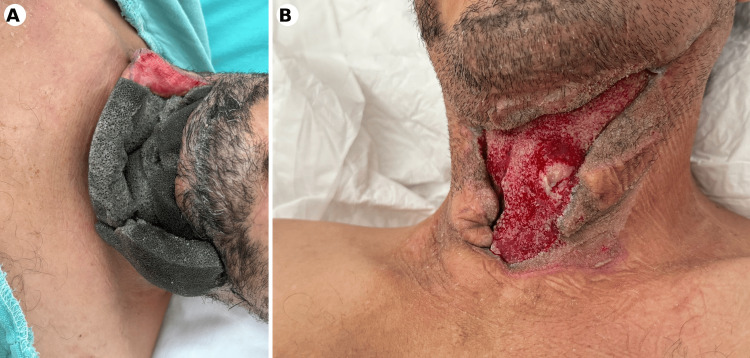
Pre-reconstructive wound status. Panel A shows a functional NPWT/VAC system applied to the anterior cervical region, demonstrating hemopurulent output secondary to retained sponge material and superinfection with *Pseudomonas aeruginosa*. Panel B shows the extensive cervicofacial soft tissue defect following VAC removal, demonstrating a viable granulation tissue bed after control of bilateral necrotizing fasciitis. All images were published with the patient's written informed consent. NPWT: negative pressure wound therapy; VAC: vacuum-assisted closure

Nine days after VAC removal, re-evaluation by the Oral and Maxillofacial Surgery service identified residual VAC sponge material retained within the surgical wound bed. Approximately 30 mL of frankly purulent material was drained, surgical irrigation was performed, a wound culture was obtained, and the VAC system was reapplied (Figure 1). Culture results reported the growth of *Pseudomonas aeruginosa*. Laboratory studies obtained three days prior had documented resolution of the leukocytosis (white blood cell count 6,700 cells/mm³), hemoglobin of 10 g/dL, creatinine of 0.7 mg/dL, and coagulation parameters within acceptable limits (PT, 16.4 sec; INR, 1.28), collectively supporting the planning of the reconstructive procedure.

**Figure 2:**
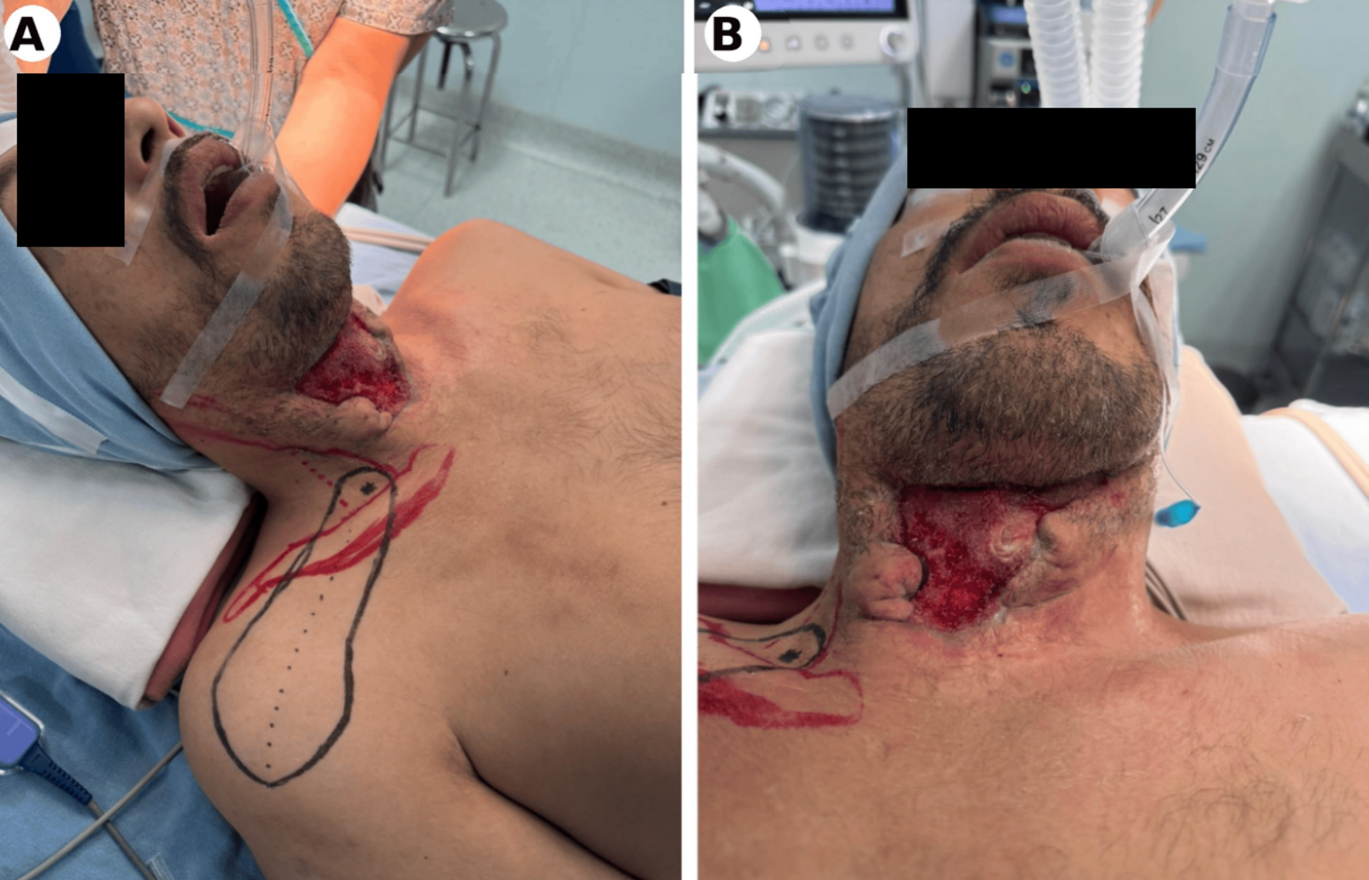
Preoperative planning of the pedicled supraclavicular fasciocutaneous flap A) Surface markings indicating the supraclavicular artery pedicle axis (dotted line) and planned skin paddle outline, with the cervicofacial defect visible. (B) Intraoperative view of the recipient defect with flap markings visible on the chest prior to flap elevation and transfer.

Once the infectious process was controlled, and given the presence of an extensive cervicofacial defect, reconstruction was planned by our Plastic and Reconstructive Surgery service using a pedicled supraclavicular flap (Figure 2).

**Figure 3:**
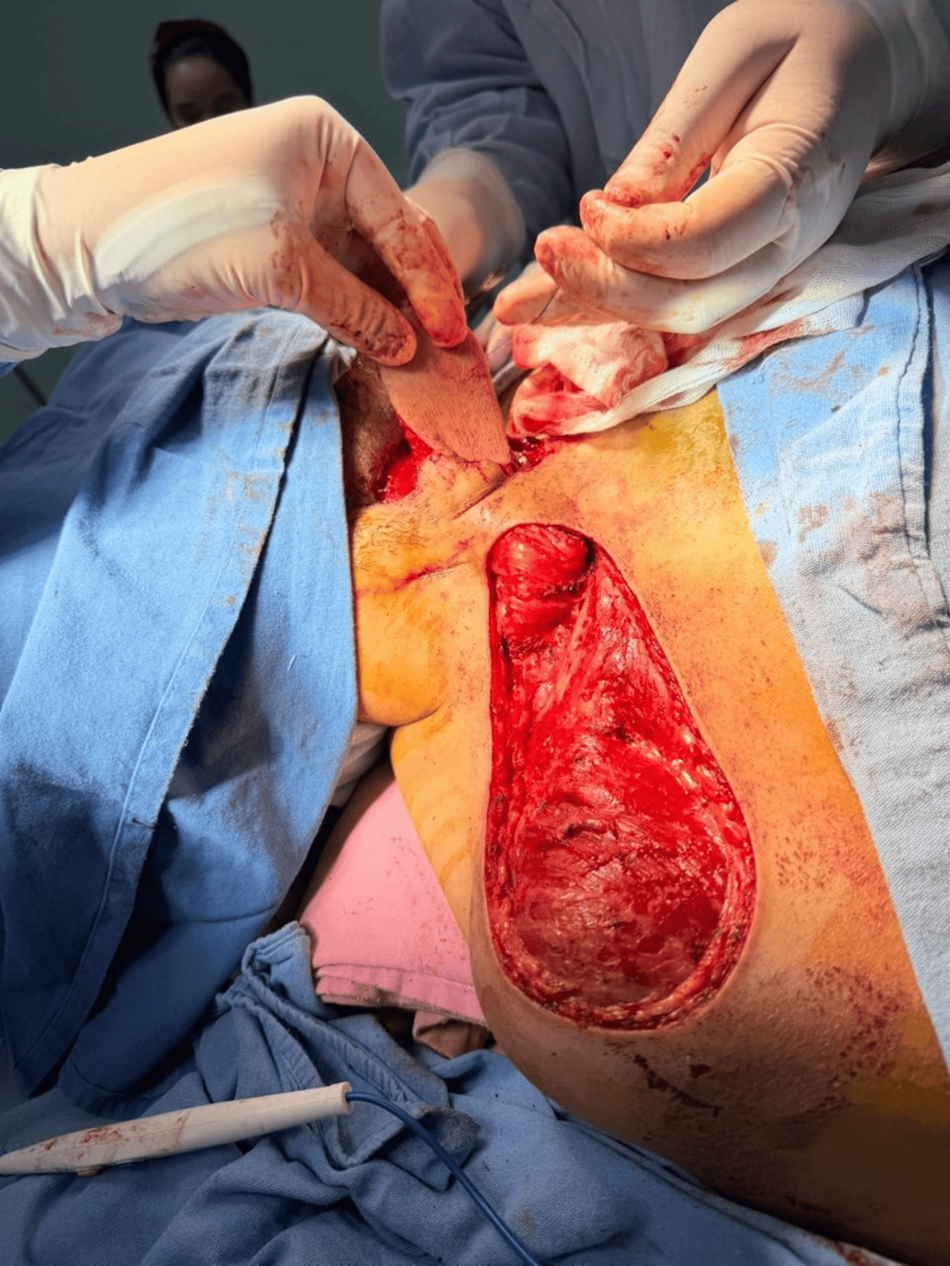
Intraoperative elevation of the supraclavicular fasciocutaneous flap Intraoperative elevation of the right supraclavicular fasciocutaneous flap in the subfascial plane, demonstrating identification of the supraclavicular artery pedicle and the harvested skin paddle prior to subcutaneous tunneling toward the cervicofacial recipient site. The tunneling step was performed through a subcutaneous passage created between the donor site and the defect, allowing transposition of the flap without cutaneous incision of the intervening skin bridge.

Under general anesthesia, with the patient in dorsal decubitus at 45 degrees, the right supraclavicular and deltoid regions were marked, and standard aseptic preparation and sterile draping were performed. The incision began in the deltoid region following the preoperative markings, with identification of the anterolateral fascia of the deltoid muscle. The fasciocutaneous flap was elevated in a subfascial plane from lateral to medial until reaching the supraclavicular region, where dissection continued in a subperiosteal plane, allowing identification of the vascular pedicle dependent on the supraclavicular artery, which was circumferentially dissected in the subfascial plane to achieve complete pedicle release (Figure 3).

**Figure 4:**
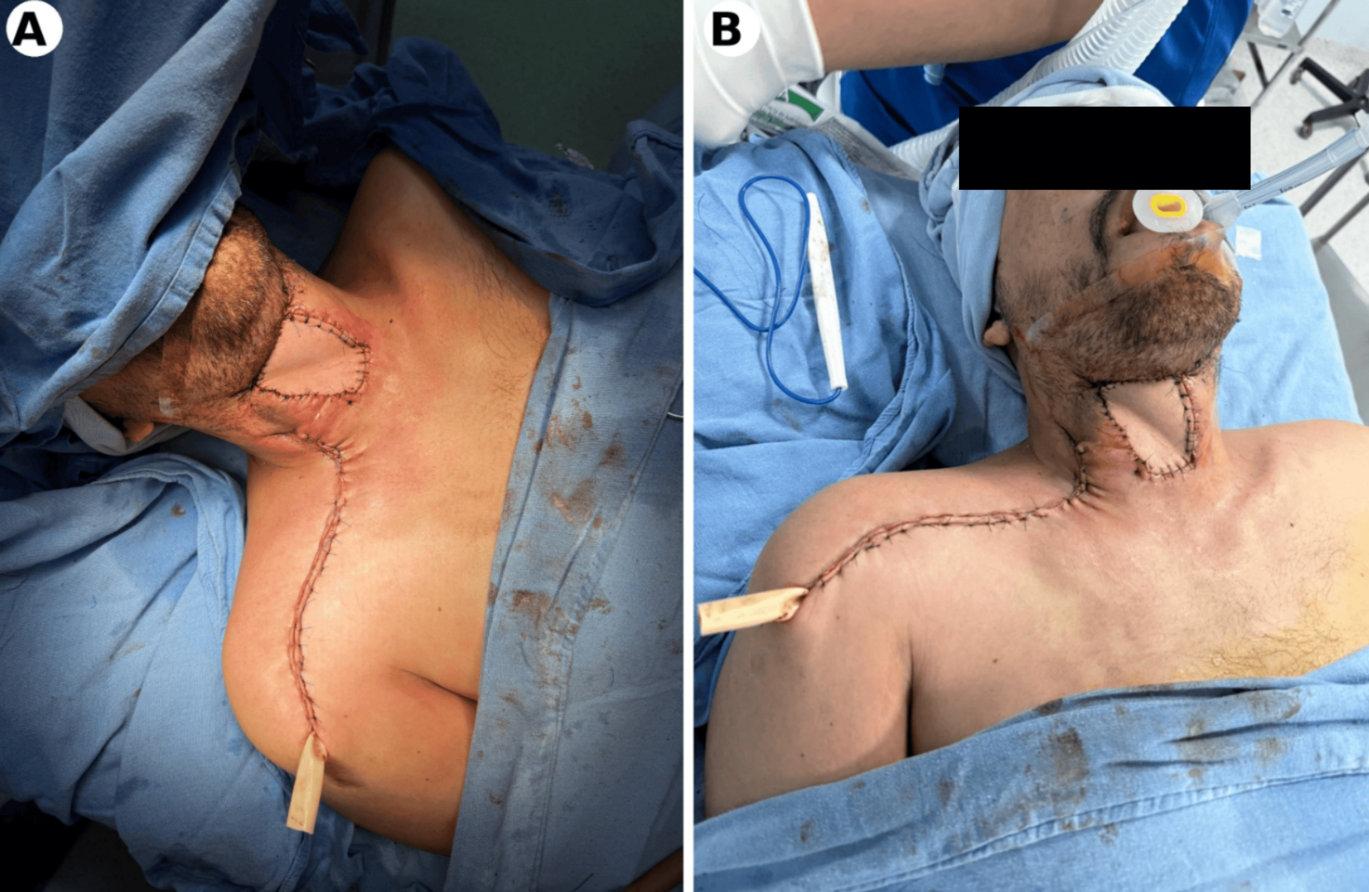
Immediate postoperative result following supraclavicular flap reconstruction (A) Close-up view of the flap inset at the cervicofacial recipient site with nylon 4-0 suture fixation and Penrose drain at the donor site. (B) Panoramic view demonstrating flap coverage of the cervicofacial defect and primary closure of the donor site extending to the deltoid region.

**Figure 5:**
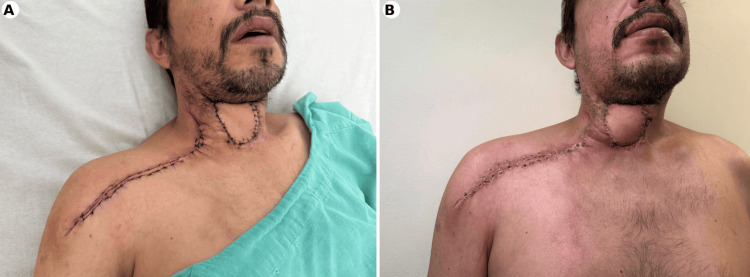
Postoperative follow-up after cervicofacial reconstruction with supraclavicular flap (A) Early in-hospital follow-up showing adequate flap integration and initial healing of recipient and donor sites. (B) Outpatient follow-up demonstrating advanced flap healing, donor site scar maturation, and satisfactory cervicofacial contour restoration (lateral view).

The recipient site was prepared with aseptic technique, with wound edge remodeling and soft tissue release using the undermining technique. A subcutaneous tunnel of approximately 2 inches was created between the donor and recipient sites. The distal skin paddle was designed according to the defect dimensions (7 × 5 cm), and the proximal end of the flap was de-epithelialized. The flap was tunneled to the recipient site and secured to the cutaneous margins with 4-0 nylon suture. Hemostasis was confirmed, and the donor site was closed in layers with placement of a ½-inch Penrose drain. Flap viability was confirmed by puncture, with adequate coloration and temperature observed throughout. The procedure was completed without complications (Figure 4). The patient demonstrated favorable evolution in the immediate postoperative period without complications (Figure 5).

## Discussion

Odontogenic infections represent the most frequent etiology of cervical necrotizing fasciitis, accounting for up to 47% of reported cases in the literature [2]. It is important to note, however, that progression from an odontogenic abscess to necrotizing fasciitis is an uncommon complication; in the majority of cases, infection remains confined to a localized cervical abscess. When spread does occur, it propagates through the fascial spaces of the neck, facilitating extension toward vital structures and even the mediastinum. The present case illustrates precisely this progression: despite the presence of factors associated with adverse outcomes, including delayed presentation and hemodynamic compromise on admission, a favorable result was achieved. This outcome is attributable to timely surgical decision-making, broad-spectrum antimicrobial coverage, and the adjunctive use of NPWT, consistent with evidence supporting an aggressive, multidisciplinary protocol as a critical determinant of survival in this entity [11].

In the present case, the combination of marked leukocytosis (26,190 cells/mm³), elevated C-reactive protein (16.4 mg/dL), rapidly progressing cervical edema, and systemic compromise raised early suspicion of a necrotizing process, prompting urgent surgical exploration. The LRINEC score was applied as an adjunctive diagnostic tool to help distinguish necrotizing fasciitis from other severe soft tissue infections. When applied specifically to the head and neck region, a systematic review and meta-analysis have reported a diagnostic odds ratio of 13.99 for a cutoff value of 6, supporting its utility as a complementary parameter in this anatomical region [5]. Although clinical judgment remains the cornerstone of diagnosis, the integration of objective laboratory parameters such as those observed in this patient reinforces the importance of early recognition in guiding timely surgical decision-making.

Standard management of cervical necrotizing fasciitis consists of broad-spectrum antimicrobial therapy, aggressive surgical debridement, and intensive care unit support [11]. In the present case, wound cultures yielded growth of *Pseudomonas aeruginosa*, guiding targeted antimicrobial adjustment. Following a single surgical debridement, NPWT was applied, facilitating exudate control, reduction of bacterial burden, and promotion of granulation tissue formation prior to definitive reconstruction. This approach is consistent with reported series in the head and neck region, where NPWT applied after debridement has been associated with reduced operative frequency, decreased inflammatory markers, and accelerated wound healing [12]. Of note, the short duration of VAC therapy required in this case reflects adequate wound bed preparation achieved after a single debridement, a finding that aligns with evidence suggesting that early NPWT application following complete necrosectomy may limit the need for repeated surgical interventions in selected patients [12].

Following infection control and adequate wound bed preparation with NPWT, definitive reconstruction was performed in a delayed fashion using a pedicled supraclavicular artery island flap. This flap represents an increasingly favored option for cervicofacial reconstruction due to its wide arc of rotation, close color and texture match to the head and neck skin, fasciocutaneous composition, and minimal donor site morbidity [8,9]. In the present case, the extent of tissue loss following debridement measured approximately 15 cm, a defect size well within the reconstructive capacity of the supraclavicular flap, which can be harvested up to 35 cm in length [9]. The delayed timing of reconstruction allowed confirmation of infection eradication prior to flap transfer, reducing the risk of flap compromise in a previously contaminated field. Its reliability in patients with significant comorbidities, straightforward dissection, and the absence of microsurgical requirements make it a practical first-line reconstructive option in this challenging clinical scenario [8].

The selection of a fasciocutaneous flap over a split-thickness skin graft for cervical reconstruction in this case was based on well-established wound biology principles. Skin grafts are fundamentally dependent on recipient bed vascularity for successful engraftment, rendering them unreliable in previously infected or structurally compromised wound beds [13]. In contrast, flaps carry an intrinsic vascular supply independent of the recipient site, making them the preferred option when wound bed quality is suboptimal [13]. Beyond biological reliability, flaps provide superior functional and aesthetic outcomes in the cervical region: they restore three-dimensional contour and tissue bulk, resist contraction during healing, a particularly relevant concern given the mobility demands of the neck, and achieve a closer color and texture match to native cervical skin compared to grafts [13]. Furthermore, well-vascularized flap tissue has demonstrated enhanced capacity to clear residual bacterial burden and resist reinfection in previously infected surgical fields, properties that skin autografts cannot provide [14].

The decision to perform pedicled rather than free flap reconstruction was deliberate and context-specific. While microsurgical free tissue transfer remains the gold standard for large composite head and neck defects in high-resource centers, its execution requires an operating microscope, microvascular instrumentation, specialized surgical training, and ICU-level postoperative flap monitoring infrastructure, resources that were not available at our institution [15]. This clinical reality is widely recognized in the reconstructive literature, and guidelines for low-resource settings explicitly recommend regional flaps, including the supraclavicular flap, as the primary reconstructive option when free flap infrastructure cannot be guaranteed [16]. Beyond infrastructure considerations, the pedicled supraclavicular flap offers objective perioperative advantages: a systematic review comparing the supraclavicular artery island flap to free tissue transfer demonstrated a large-effect-size reduction in operative time, favoring the pedicled approach, with comparable rates of total flap loss, partial necrosis, and wound dehiscence [17]. Additionally, a direct comparative analysis reported significantly lower total hospital charges and shorter operative times without differences in wound healing outcomes [18]. These data collectively support the supraclavicular pedicled flap as an evidence-based, reliable, and resource-appropriate reconstructive choice in the present clinical context.

Preoperatively, the supraclavicular artery and vein were identified using a handheld Doppler ultrasound probe, and the pedicle was marked accordingly. The patient was positioned in dorsal decubitus at a 45-degree angle. The incision was carried from the marked pedicle site to the deltoid muscle, and the flap was elevated in a subfascial plane from lateral to medial. Perforating vessels from the deltoid muscle were divided, and the pedicle was identified in the middle third of the flap by transillumination. Upon reaching the medial limit of the incision at the pedicle origin, the skin was incised superficially with careful preservation of the supraclavicular vessels. The pedicle was then prepared subcutaneously in its superior aspect and at a subfascial level inferiorly, after which the flap was tunneled to the recipient site. The donor site was closed primarily with subcutaneous sutures without the need for skin grafting, even given the extent of the harvest [8].

The present case underscores the value of a sequential, protocol-driven approach to cervicofacial necrotizing fasciitis, in which timely surgical debridement, targeted antimicrobial therapy, adjunctive NPWT, and staged reconstruction constitute complementary steps toward a favorable outcome. The uncomplicated postoperative course and satisfactory wound healing observed in this patient support the feasibility of the supraclavicular pedicled flap as a reliable reconstructive option following infection control in this setting. The integration of NPWT as a bridge to reconstruction proved particularly valuable, facilitating wound bed preparation and enabling definitive closure without the need for repeated debridements. As with all case reports, the principal limitation of this study is the inability to draw generalizable conclusions from a single patient experience. Additionally, the absence of a comparative group precludes formal assessment of outcomes relative to alternative reconstructive strategies. Nevertheless, this case contributes to the growing body of evidence supporting the combined use of NPWT and supraclavicular flap reconstruction as an effective and reproducible protocol for the management of extensive cervicofacial defects resulting from necrotizing fasciitis, particularly in centers where microsurgical resources may be limited.

## Conclusions

Cervicofacial necrotizing fasciitis is a life-threatening surgical emergency whose prognosis depends on early diagnosis and aggressive treatment. The multimodal approach, consisting of broad-spectrum antibiotic therapy, early debridement, and negative pressure therapy, enabled infection control and progressive preparation of the wound bed for definitive reconstruction.

The choice of the pedicled supraclavicular flap over a split-thickness skin graft was based on the need for three-dimensional coverage with an intrinsic vascular supply over exposed noble structures, in a previously infected bed where graft viability would have been unreliable. The pedicled modality was preferred over free microvascular transfer due to its consistent pedicle, generous arc of rotation, and absence of anastomotic requirements, advantages that translate into shorter operative times and reduced anesthetic risk in a patient with a compromised general status following sepsis. Additionally, institutional constraints precluded the routine use of free flaps, further reinforcing the selection of a technically reproducible and resource-appropriate reconstructive option.

Acknowledged limitations include the absence of formal severity scoring at admission and the lack of standardized long-term functional follow-up, both inherent to the nature of a case report. This work contributes, nonetheless, to documenting a safe and reproducible reconstructive strategy for complex postinfectious cervicofacial defects and provides a foundation for future case series evaluating functional and quality-of-life outcomes.
